# Supplementary material for: Determining the perceived acceptability of an intervention designed to improve health literacy around developmentally appropriate play during infancy, with a community advisory group of mothers, in Soweto, South Africa
Source: PLOS Glob Public Health. 2024 Aug 29;4(8):e0002233. doi: 10.1371/journal.pgph.0002233 (PMC11361429; doi:10.1371/journal.pgph.0002233)
Supplement: S1 Appendix — (PDF) [file pgph.0002233.s001.pdf]

Appendix 1: Participant breakdown for each Round of Data collection

Round 1 (N = 20)

FGD 1
n=8

FG2
n=6

FGD 3
n=6

Round 2 (N=15) (Two weeks after Round 1)

FGD 1
n=7

FGD 2
n=8

Round 3 (N=14) (One week after Round 2)

Telephonic questionnaire
n=14
