# Supplementary material for: Determining the perceived acceptability of an intervention designed to improve health literacy around developmentally appropriate play during infancy, with a community advisory group of mothers, in Soweto, South Africa
Source: PLOS Glob Public Health. 2024 Aug 29;4(8):e0002233. doi: 10.1371/journal.pgph.0002233 (PMC11361429; doi:10.1371/journal.pgph.0002233)
Supplement: S2 Appendix — (PDF) [file pgph.0002233.s002.pdf]

Appendix 1: Focus Group Discussion 1 (Round 1) Photos

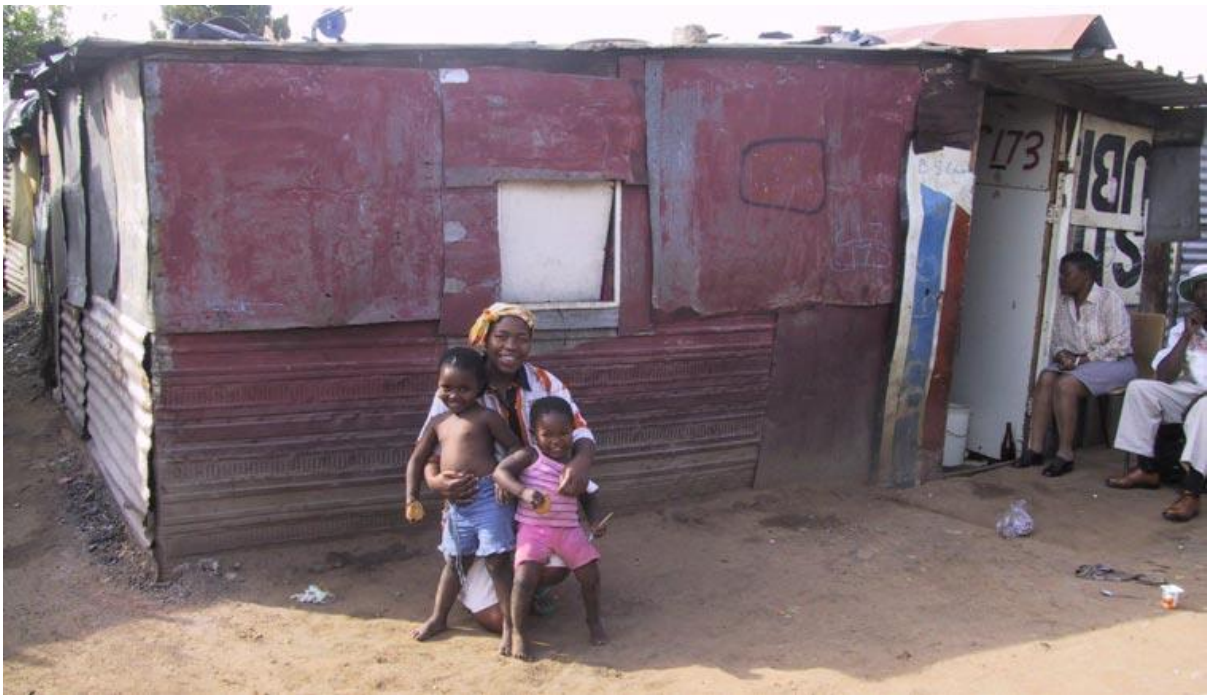

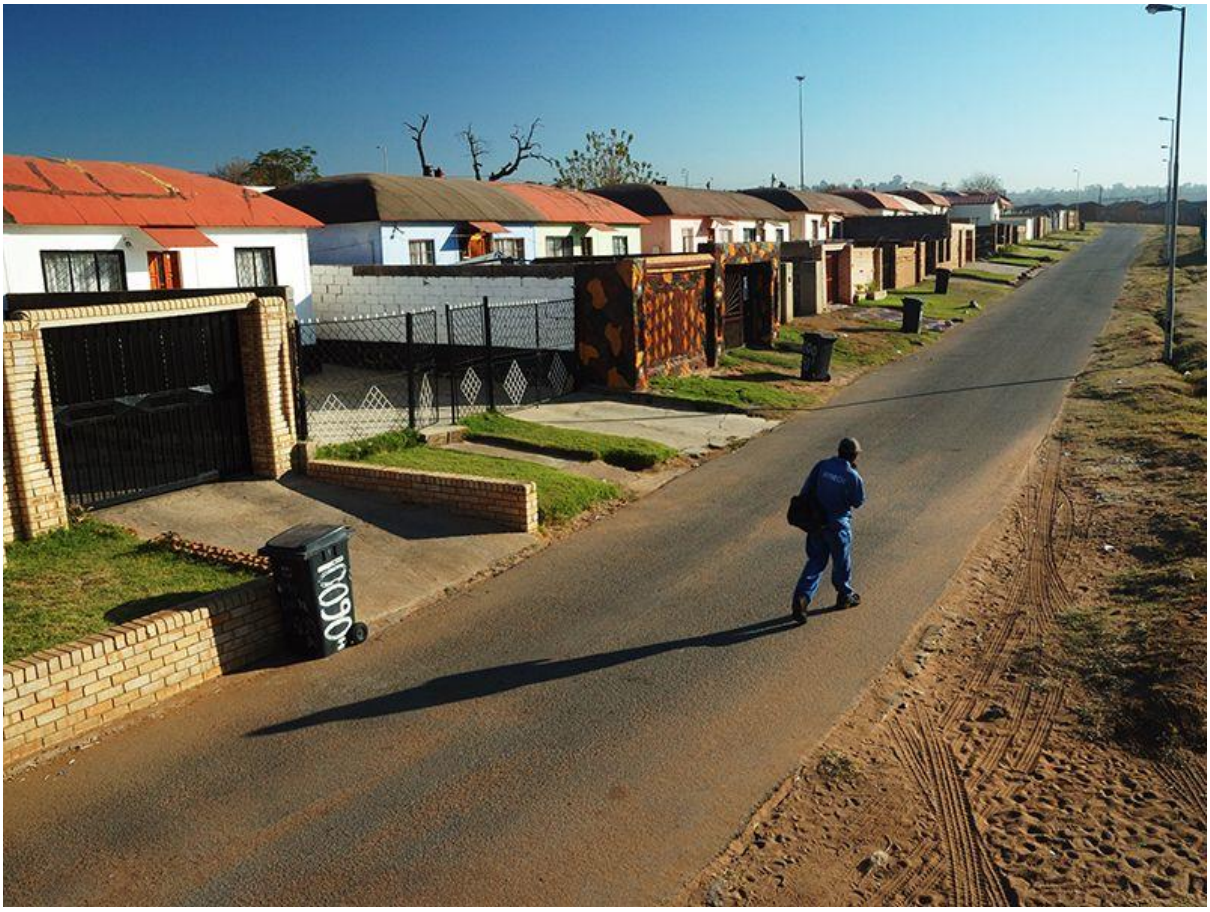

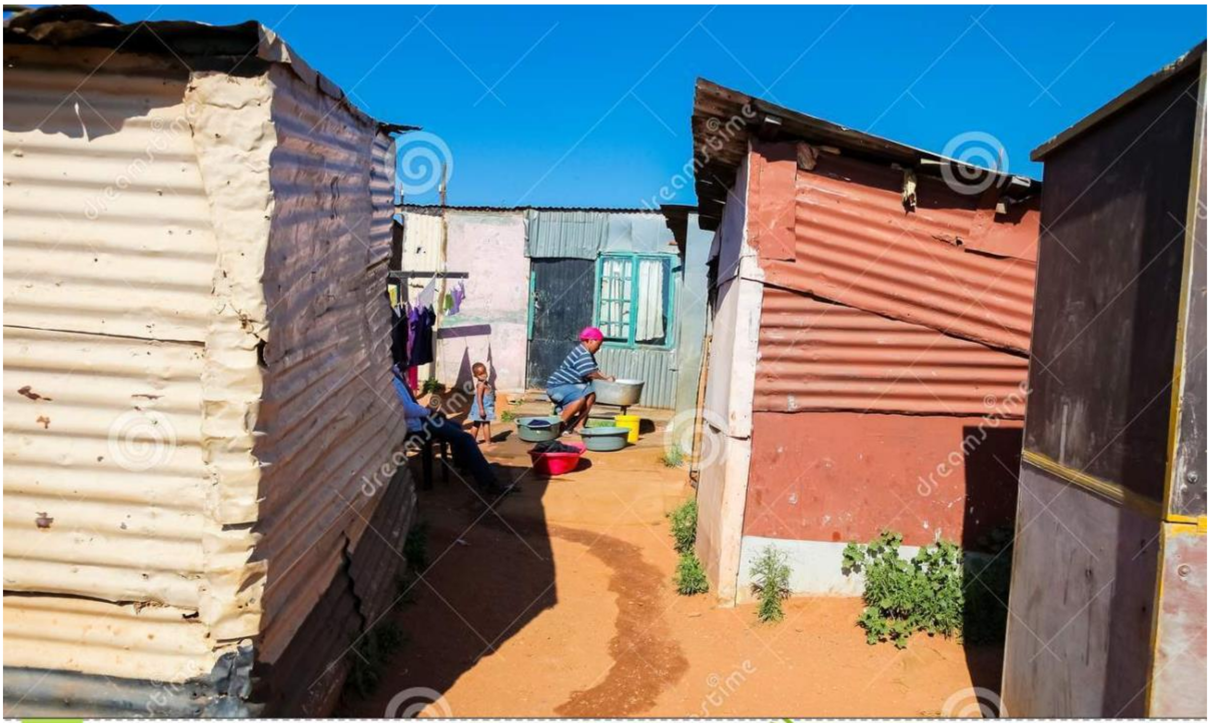

Download from
Dreamstime.com
This watermarked comp image is for previewing purposes only.
ID 121953329
© Michael Turner | Dreamstime.com

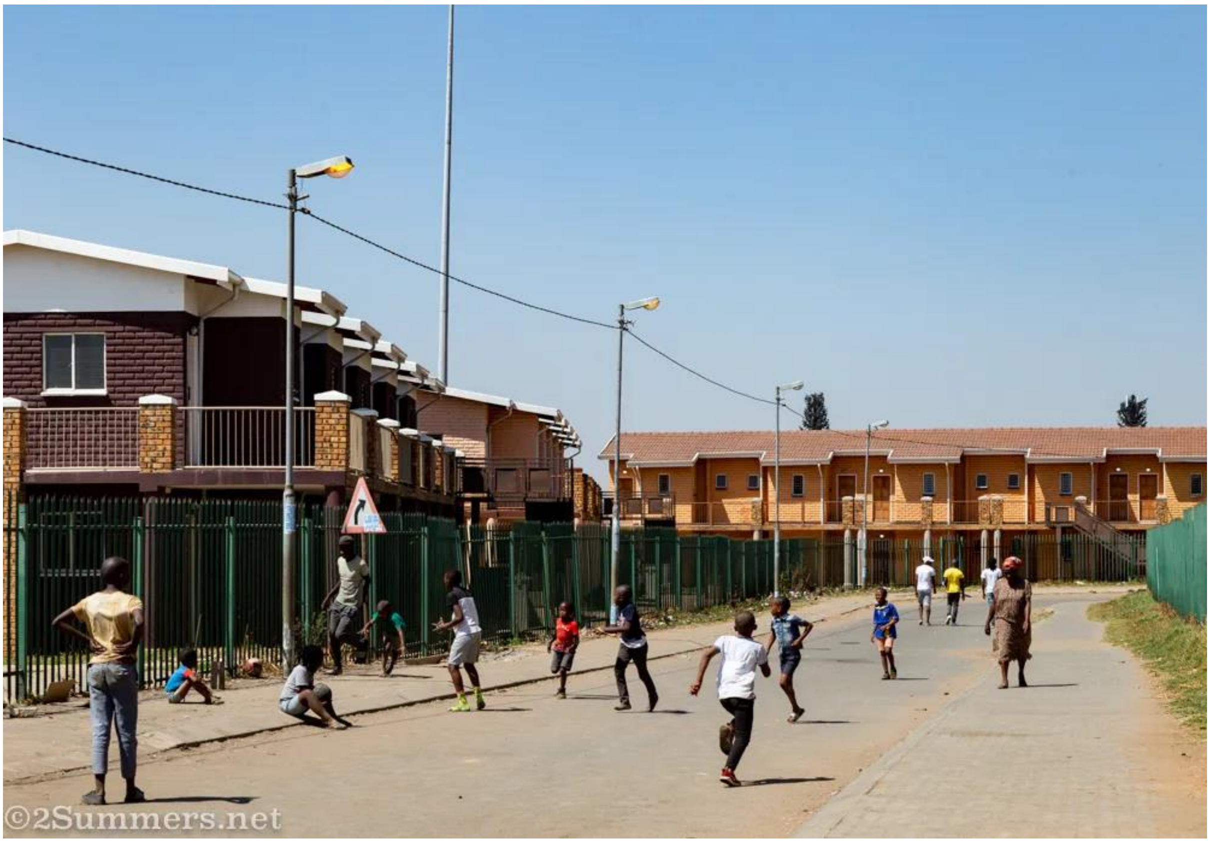

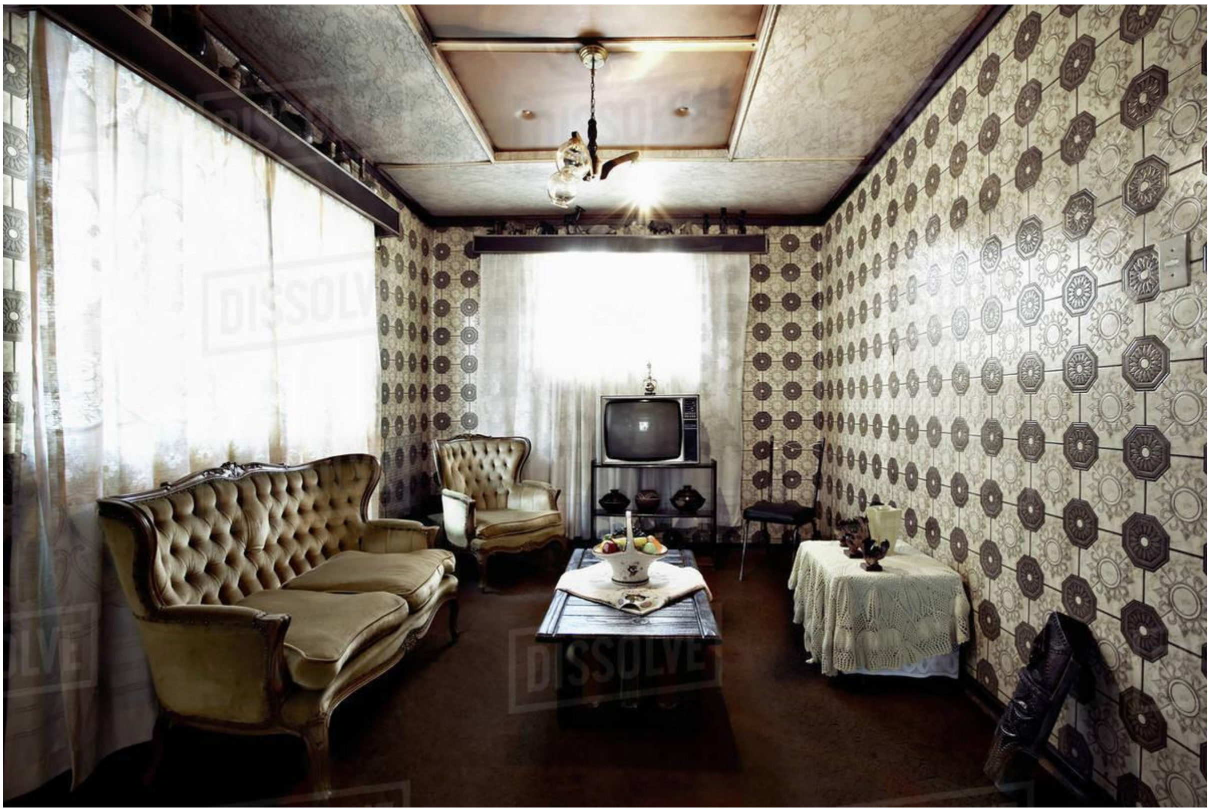
DISSOLVE

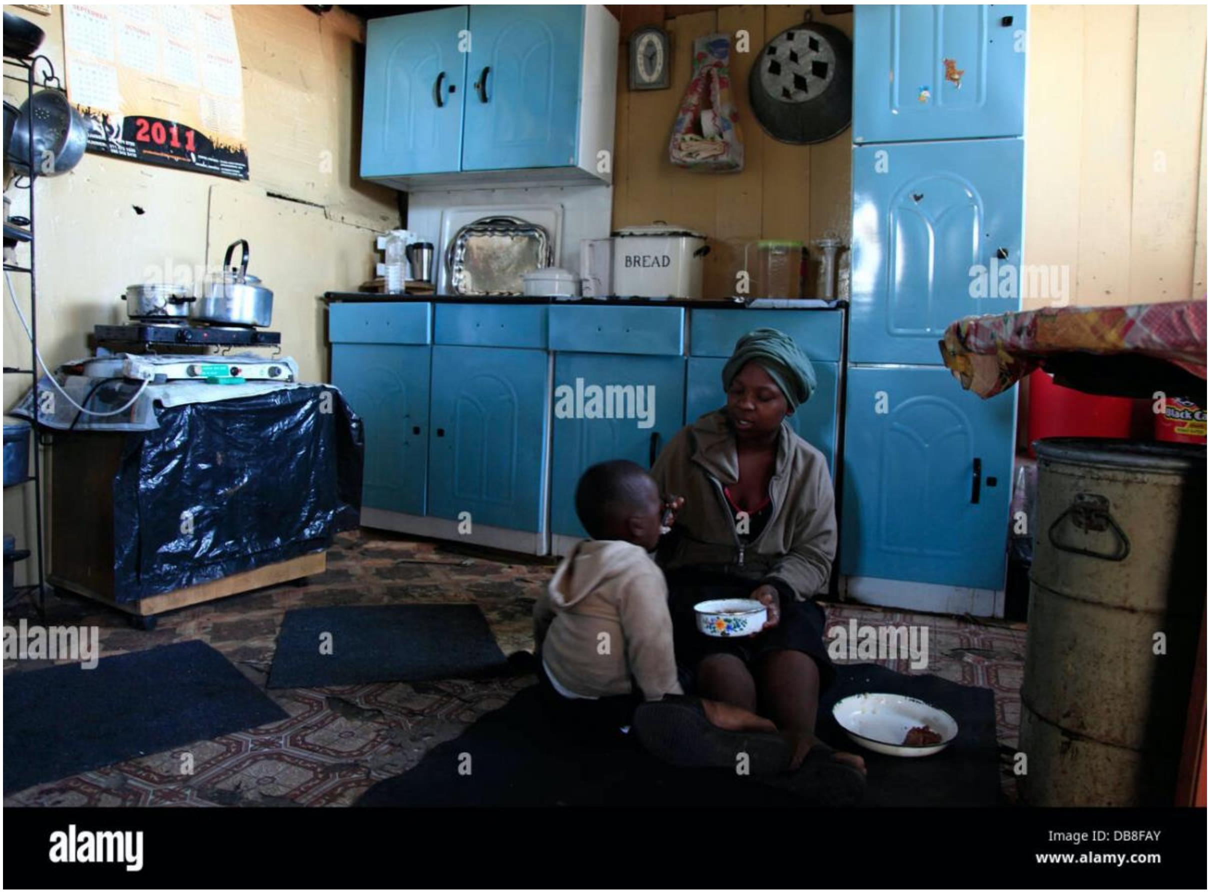
2011
BREAD
alamy
Image ID: DB8FAY
www.alamy.com
