# Supplementary material for: Determining the perceived acceptability of an intervention designed to improve health literacy around developmentally appropriate play during infancy, with a community advisory group of mothers, in Soweto, South Africa
Source: PLOS Glob Public Health. 2024 Aug 29;4(8):e0002233. doi: 10.1371/journal.pgph.0002233 (PMC11361429; doi:10.1371/journal.pgph.0002233)
Supplement: S3 Appendix — (PDF) [file pgph.0002233.s003.pdf]

Acceptability Questionnaire (CAG) (Telephonic)

CAG Participant ID: ________________________

Date: ________________________

Age of child: ________________________

In your last Focus Group Discussion, you were asked to go home and play some of the games and make a sensory square and/or the shaker, which were discussed in the group.

We are going to ask you a few questions about your experience at home. There is no right or wrong answer and it's fine if you didn't manage to do as much as you would have liked but please do be as honest as possible as your answers will help us to make the activities as easy as possible to use with other mothers in future.

1. Which of the activities did you manage to do at home?

1.a. Sensory Square	Yes/ no
1.b. Shaker	Yes/no
1.c. Game 1 – whisper a secret	Yes/no
1.d. Game 2 – touch and tickle	Yes/no
1.e. Game 3 – rolling around	Yes/no
1.f. Game 4 – shake some noise	Yes/no
1.g. Game 5 – trying new smells	Yes/no

2. If no to all above, why?

GAMES QUESTIONS

3. Did you and your baby enjoy engaging in the games? | yes | no |
   a. What did you like about them?
   b. What did you not like about them?

4. Did you find the games difficult or easy to do? | yes | no |
   a. Please explain

5. Did you understand how to do the games? | yes | no |
   a. Were the instructions clear? | yes | no |
      i.
   b. If no, was there anything else that could have been added to make it easier?

6. Did you have enough time to fit them into your schedule? | yes | no |
   a. If no, can explain what made it difficult to find time to do it?
   b. What would help to remind you or help you find time to include the games in your day?

7. Do you think the games are beneficial for you and the baby?

yes	no

   a. Why?

8. Were you confident that you knew how to do the activity and were doing it correctly?

yes	no

   a. How do you think you could become more confident in it?

SENSORY SQUARE + SHAKER QUESTIONS

9.a. What did you like about the square and/or shaker?

9.b. What did you not like about them?

10.a. How easy was it to find the resources that you needed to make it, in your house (or from a friend/ family member)?

Very easy	Easy	Difficult	Very difficult

10.b. Did you have to buy any of the resources?

yes	no

10.c. If yes, what did you buy?
11.a. Were the toys easy to make?<br><br>| yes | no | <br>11.b. Did you prefer having the video or watching someone show you how to make them in person?<br><br>| In person | video | <br>11.c. Why?
12.a. Did the toy-making take up a lot of your time?<br><br>| yes | no | <br>12.b. If yes, why do you think it did?
13. How often would you like us to send instructions to make a new toy?
14. Do you have any other comments/ suggestions about any of the activities we asked you to do at home?

Thank you for your time.
