# Supplementary figures and images for: Determining the perceived acceptability of an intervention designed to improve health literacy around developmentally appropriate play during infancy, with a community advisory group of mothers, in Soweto, South Africa
Source: PLOS Glob Public Health. 2024 Aug 29;4(8):e0002233. doi: 10.1371/journal.pgph.0002233 (PMC11361429; doi:10.1371/journal.pgph.0002233)

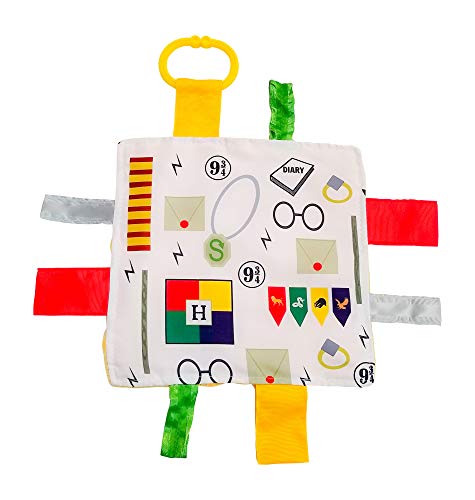

Supplement: S1 Fig — (JPEG) [file pgph.0002233.s004.jpeg]
