# Supplementary material for: Determining the perceived acceptability of an intervention designed to improve health literacy around developmentally appropriate play during infancy, with a community advisory group of mothers, in Soweto, South Africa
Source: PLOS Glob Public Health. 2024 Aug 29;4(8):e0002233. doi: 10.1371/journal.pgph.0002233 (PMC11361429; doi:10.1371/journal.pgph.0002233)
Supplement: S1 Checklist — (DOCX) [file pgph.0002233.s007.docx]

STROBE Statement—checklist of items that should be included in reports of observational studies

|  | Item No. | Recommendation | Page  No. | | | Relevant text from manuscript |  |
| --- | --- | --- | --- | --- | --- | --- | --- |
| **Title and abstract** | 1 | (*a*) Indicate the study’s design with a commonly used term in the title or the abstract | 2 | | | This mixed methods, cross-sectional study aimed to test the acceptability of an intervention developed to improve health literacy around play and development with mothers from the Soweto community in South Africa |  |
|  |  | (*b*) Provide in the abstract an informative and balanced summary of what was done and what was found | 2-3 | | | Fifteen mothers with infants aged 0-3 months were included to form a Community Advisory Group (CAG). Two rounds of focus group discussions (FGDs) were conducted to explore the understanding of, and barriers to play and development, and to determine the acceptability of an intervention prototype presented to the participants in the group. The prototype included developmentally appropriate activities presented in video format, demonstrations of how to make toys, as well as infographics and other educational material which would be delivered to participants as part of the intervention. A further questionnaire was administered to participants one week after the focus group to determine the acceptability of intervention activities which the participants engaged with at home, with their infants. A major theme emerging from the FGDs was around the reported barriers to play, which included limited options for safe outdoor play, overcrowding, insufficient time, limited resources and conflicting information. Participants suggested that the intervention content be delivered every 1-2 weeks, through a data-free app, in English with subtitles in the local language wherever possible. Overall, the prototype activities tested at home were deemed acceptable using the seven constructs of acceptability. The reported barriers, together with a lack of motivation and self-efficacy observed in the participants, guided the researchers to develop intervention content focused on improving health literacy in play and development, delivered in the form of an interactive mobile app |  |
| Introduction | | | | |  | | |
| Background/rationale | 2 | Explain the scientific background and rationale for the investigation being reported | 3-5 | Please see introduction section | | | |
| Objectives | 3 | State specific objectives, including any prespecified hypotheses | 5 | In light of the above, this study aimed to test the acceptability of an intervention developed to improve health literacy around play and development with mothers from the Soweto community in South Africa. The researchers hypothesized that increased health literacy in mothers will improve capability, motivation and opportunity, thereby ultimately resulting in greater participation in infant play. The specific objectives in this paper were:   1. To understand the challenges and barriers that a group of Soweto mothers experience when facilitating play with their young infants. 2. To present a prototype of intervention activities and content, discuss perceptions as to how the activities and content may improve health literacy (thereby encouraging participation in play), and how best to present the intervention. 3. To determine the acceptability of the intervention prototype after being tested by the mothers in Soweto. | | | |
| Methods | | | | |  | | |
| Study design | 4 | Present key elements of study design early in the paper | 7 | Please see 2.2. Data collection | | | |
| Setting | 5 | Describe the setting, locations, and relevant dates, including periods of recruitment, exposure, follow-up, and data collection | 6-9 | Please see 2.2. Data collection, “Round 1”, “Round 2” and “Round 3” | | | |
| Participants | 6 | (*a*) *Cohort study*—Give the eligibility criteria, and the sources and methods of selection of participants. Describe methods of follow-up  *Case-control study*—Give the eligibility criteria, and the sources and methods of case ascertainment and control selection. Give the rationale for the choice of cases and controls  *Cross-sectional study*—Give the eligibility criteria, and the sources and methods of selection of participants | 6-7 | See 2.1 Study setting and participants | | | |
|  |  | (*b*) *Cohort study*—For matched studies, give matching criteria and number of exposed and unexposed  *Case-control study*—For matched studies, give matching criteria and the number of controls per case |  |  | | | |
| Variables | 7 | Clearly define all outcomes, exposures, predictors, potential confounders, and effect modifiers. Give diagnostic criteria, if applicable | 6 | The primary outcome is acceptability of the prototype | | | |
| Data sources/ measurement | 8* | For each variable of interest, give sources of data and details of methods of assessment (measurement). Describe comparability of assessment methods if there is more than one group | *8-9* | Acceptability was measured using the seven constructs as outlined by Sekhon et al., (2017)  All participants started the session by providing socio-demographic information in a questionnaire. | | | |
| Bias | 9 | Describe any efforts to address potential sources of bias | 10; 28-29 | As the study started with qualitative data collection, there is a small sample size, and therefore the authors recognise that this is not representative of the general population. Efforts to reduce bias are described in the analysis section as well as under the limitations section. | | | |
| Study size | 10 | Explain how the study size was arrived at | 8 | As the study started with qualitative data collection, the aim was to reach data saturation. We therefore started with three focus groups in round 1, then two focus groups in round 2 and then stopped recruiting as no new themes were presented. The quantitative study size was based on the number of participants in the Focus Group Discussion. | | | |

Continued on next page

| Quantitative variables | 11 | Explain how quantitative variables were handled in the analyses. If applicable, describe which groupings were chosen and why | 10 | The data was purely descriptive. Means and standard deviations or percentages were calculated on Microsoft Excel and used to describe the demographics of the participants as well as quantitative data from the acceptability questionnaire. |
| --- | --- | --- | --- | --- |
| Statistical methods | 12 | (*a*) Describe all statistical methods, including those used to control for confounding |  | Data was purely descriptive.  Means and standard deviations or percentages were calculated on Microsoft Excel and used to describe the demographics of the participants as well as quantitative data from the acceptability questionnaire. |
|  |  | (*b*) Describe any methods used to examine subgroups and interactions |  | N/A |
|  |  | (*c*) Explain how missing data were addressed | 19 | There were no missing data in the completed questionnaires, however one participant did not complete a questionnaire, and therefore the authors only analysed complete data (we excluded the participant who didn’t have full data |
|  |  | (*d*) *Cohort study*—If applicable, explain how loss to follow-up was addressed  *Case-control study*—If applicable, explain how matching of cases and controls was addressed  *Cross-sectional study*—If applicable, describe analytical methods taking account of sampling strategy |  | N/A |
|  |  | (*e*) Describe any sensitivity analyses |  | N/A |
| Results | | | | |
| Participants | 13* | (a) Report numbers of individuals at each stage of study—eg numbers potentially eligible, examined for eligibility, confirmed eligible, included in the study, completing follow-up, and analysed | 19 | One participant was not able to be contacted and therefore only fourteen participants completed the telephonic questionnaire. The number of participants completing each respective activity is represented in Fig 3. The discrepancy in number of mothers completing the activities is due to the mothers only being instructed to pick a few activities to complete at home (i.e. they did not have to complete all of them in the week). |
|  |  | (b) Give reasons for non-participation at each stage | 19 | One participant was not able to be contacted. |
|  |  | (c) Consider use of a flow diagram |  | N/A |
| Descriptive data | 14* | (a) Give characteristics of study participants (eg demographic, clinical, social) and information on exposures and potential confounders | 10-11 |  |
|  |  | (b) Indicate number of participants with missing data for each variable of interest |  | N/A |
|  |  | (c) *Cohort study*—Summarise follow-up time (eg, average and total amount) | N/A |  |
| Outcome data | 15* | *Cohort study*—Report numbers of outcome events or summary measures over time | *N/A* |  |
|  |  | *Case-control study—*Report numbers in each exposure category, or summary measures of exposure | *N/A* |  |
|  |  | *Cross-sectional study—*Report numbers of outcome events or summary measures | *19-23* |  |
| Main results | 16 | (*a*) Give unadjusted estimates and, if applicable, confounder-adjusted estimates and their precision (eg, 95% confidence interval). Make clear which confounders were adjusted for and why they were included | N/A |  |
|  |  | (*b*) Report category boundaries when continuous variables were categorized | N/A |  |
|  |  | (*c*) If relevant, consider translating estimates of relative risk into absolute risk for a meaningful time period | N/A |  |

Continued on next page

| Other analyses | 17 | Report other analyses done—eg analyses of subgroups and interactions, and sensitivity analyses | N/A |  |
| --- | --- | --- | --- | --- |
| Discussion | | | | |
| Key results | 18 | Summarise key results with reference to study objectives | 23-28 |  |
| Limitations | 19 | Discuss limitations of the study, taking into account sources of potential bias or imprecision. Discuss both direction and magnitude of any potential bias | 28 |  |
| Interpretation | 20 | Give a cautious overall interpretation of results considering objectives, limitations, multiplicity of analyses, results from similar studies, and other relevant evidence | 29 | Conclusion |
| Generalisability | 21 | Discuss the generalisability (external validity) of the study results | 28 |  |
| Other information | |  | | |
| Funding | 22 | Give the source of funding and the role of the funders for the present study and, if applicable, for the original study on which the present article is based | 34 |  |

*Give information separately for cases and controls in case-control studies and, if applicable, for exposed and unexposed groups in cohort and cross-sectional studies.

**Note:** An Explanation and Elaboration article discusses each checklist item and gives methodological background and published examples of transparent reporting. The STROBE checklist is best used in conjunction with this article (freely available on the Web sites of PLoS Medicine at http://www.plosmedicine.org/, Annals of Internal Medicine at http://www.annals.org/, and Epidemiology at http://www.epidem.com/). Information on the STROBE Initiative is available at www.strobe-statement.org.
