# Supplementary material for: Determining the perceived acceptability of an intervention designed to improve health literacy around developmentally appropriate play during infancy, with a community advisory group of mothers, in Soweto, South Africa
Source: PLOS Glob Public Health. 2024 Aug 29;4(8):e0002233. doi: 10.1371/journal.pgph.0002233 (PMC11361429; doi:10.1371/journal.pgph.0002233)
Supplement: Attachment — Submitted filename: COMMENTS_Acceptability article 01 June 2023 PLOS global health.docx [file pgph.0002233.s008.docx]

Determining the perceived acceptability of an intervention designed to improve health literacy around developmentally appropriate play during infancy, with a community advisory group of mothers, in Soweto, South Africa.

Fiona Bennin^1*^, Helene Theunissen^1^, Shane A Norris^1,^ Alessandra Prioreschi^1^

^1^SA MRC/Wits Developmental Pathways for Health Research Unit, Department of Paediatrics, Faculty of Health Sciences, School of Clinical Medicine, University of the Witwatersrand, Johannesburg, South Africa;

*Corresponding author

Email: [fionabennin@gmail.com](mailto:fionabennin@gmail.com) (FB)

**Abstract**

Children require opportunities to participate in unstructured, unrestricted active play as infants, to encourage positive health, socioemotional and developmental outcomes in life. Certain social and environmental factors in the home setting can limit participation in play, particularly in low resource settings. As caregivers (particularly mothers) are their infants’ first teachers, they have the important role of providing opportunities and space for children to learn through play. This mixed methods, cross-sectional study aimed to test the acceptability of an intervention developed to improve health literacy around play and development with mothers from the Soweto community in South Africa. Fifteen mothers with infants aged 0-3 months were included to form a Community Advisory Group (CAG). Two rounds of focus group discussions (FGDs) were conducted to explore the understanding of, and barriers to play and development, and to determine the acceptability of an intervention prototype presented to the participants in the group. The prototype included developmentally appropriate activities presented in video format, demonstrations of how to make toys, as well as infographics and other educational material which would be delivered to participants as part of the intervention. A further questionnaire was administered to participants one week after the focus group to determine the acceptability of intervention activities which the participants engaged with at home, with their infants. A major theme emerging from the FGDs was around the reported barriers to play, which included limited options for safe outdoor play, overcrowding, insufficient time, limited resources and conflicting information. Participants suggested that the intervention content be delivered every 1-2 weeks, through a data-free app, in English with subtitles in the local language wherever possible. Overall, the prototype activities tested at home were deemed acceptable using the seven constructs of acceptability. The reported barriers, together with a lack of motivation and self-efficacy observed in the participants, guided the researchers to develop intervention content focused on improving health literacy in play and development, delivered in the form of an interactive mobile app. Future research will develop and test the efficacy of this intervention in a low resource setting in South Africa.

**1. Introduction**

Early childhood is one of the most important timeframes within an individual’s life, due to the rapid growth of the brain and development of the body’s systems occurring during this time [1] In particular, due to the sensitivity of the first 1000 days of life, children are receptive to a large range of environmental stimuli (both positive and negative), making it an important time for developing healthy behaviours, which encourage positive outcomes later in life [1]. One of the healthy behaviours that has an effect on the overall health of both children and adults, is physical activity. As there are numerous negative side effects of an inactive, sedentary lifestyle, these effects can take years to emerge, which makes it imperative to start encouraging physical activity as soon as infants become mobile [2]. In the early months of life, allowing infants access to unstructured, unrestrictive play at home is imperative for development and for encouraging increased physical activity [3]. This is evident in the poor developmental outcomes seen in children who spend too much time in a supine position as infants, constrained in infant seats or walkers, or in small playpens [3]. Unstructured outdoor play is particularly important as it allows space and opportunities for playful activities, which encourage development of gross motor skills (e.g. kicking and throwing balls, climbing on play equipment, etc.) and higher intensity physical activity required to meet daily movement guidelines [4]. Children need to be provided access to, and opportunities to engage in unstructured play by their caregivers, which highlights the importance of caregiver education and modelling when attempting to encourage more unstructured play with children. By focusing on increasing participation in play, infants and toddlers will naturally be more physically active, resulting in a reduction in risk of developing obesity and other non-communicable diseases later in life [5].

Early Childhood Development (ECD) refers to, “the physical, socio-emotional, cognitive, and motor development that a child experiences between birth and the age of eight” [6]. Evidence suggests that focusing on ECD has positive outcomes later in life, in the areas of language, socioemotional and cognitive development, which then positively impact an individual’s productivity in schooling and the labour market [2, 7]. The link between play and the development of motor, cognitive and socio-emotional skills is well documented [3, 8]. Infants who have delays in gross motor skill development may develop poor core strength, problems with fine motor development as well as difficulties with visual motor integration, all of which impact on academic performance and health outcomes later in life [9, 10]. Children and infants who have limited opportunities for play can struggle to connect socially with others, may develop behaviour problems and have a limited sense of self [5].

Poverty, one of the most critical determinants of childhood development, has a major impact on children’s health, specifically regarding access to quality health care, adequate housing, access to clean water and sanitation, and access to nutritious food [6]. Stunting and wasting in children under 5 years is a major public health concern requiring urgent attention in South Africa (at 23% and 3.4% respectively), as is obesity with 13% of South African children under 5 years being overweight in 2020 [11, 12]. According to the 2011 South African census, 44.5% of households qualified as no income (R0/$0) and low income households (R0-R19,200/$2800 per annum) [13]. As a result, many of these households would have no means of sending their children to formal ECD centres, which is further supported by a 2021 report which found that 64.6% of 0-4 year olds stayed at home with their parent or another adult with no involvement in, or access to ECD programmes [14].

This is important as social and environmental factors in the home setting could limit participation in play, particularly in low resource settings such as South Africa. Examples of environmental factors include outside areas which are not considered safe to play (e.g., exposure to violence, heavy traffic, pollution, unsanitary conditions, etc.) as well as inside areas which may be very small or overcrowded with no limitations on screen time and other similar sedentary behaviours [15]. Social conditions that impact play include lack of resources available for toys or books, low maternal health literacy, and poor maternal mental health [15] [16]. Former studies conducted in a low income community in South Africa found that although mothers understood the need for play in promoting development, they often lacked the incentive and motivation, as well as the knowledge of how to promote development. Many families did not read to their children or own a book; and there was often a misconception that expensive toys are needed to encourage play and development [4, 15]. Mothers reported that the main barriers to play included lack of financial resources and concerns around environmental factors such as lack of safety, fear of abduction and other structural barriers, which prevented them from encouraging children to play outside [8,15].

As caregivers are their infants’ first teachers, they have the important role of providing opportunities and space for children to learn through play [8]. Empowerment and provision of support for caregivers is therefore essential, to allow facilitation of playful learning in day-to-day activities in the home environment and community [8]. This is particularly important in a setting such as South Africa, where caregivers have expressed a lack of motivation and knowledge in how to promote development, particularly through play. Therefore, increasing caregiver health literacy is key to encouraging an increase in motivation and capability as well as a greater understanding of the importance of play and development in the early months.

For individuals to have adequate health literacy levels, they need to be able to access, understand, appraise, remember and use the information available to them [17]. It is also important to note that people learn in different ways and therefore require information to be presented in a variety of forms [18]. Furthermore, in South Africa in 2018, it was found that 43.1% of children lived with only their mothers, 33.8% with both their parents and 19.8% lived with neither parents [19]. Given the limited availability of present fathers in the South African context, the researchers chose to specifically focus on changing the behaviour of mothers.

In light of the above, this study aimed to test the acceptability of an intervention developed to improve health literacy around play and development with mothers from the Soweto community in South Africa. The researchers hypothesized that increased health literacy in mothers will improve capability, motivation and opportunity, thereby ultimately resulting in greater participation in infant play. The specific objectives in this paper were:

1. To understand the challenges and barriers that a group of Soweto mothers experience when facilitating play with their young infants.
2. To present a prototype of intervention activities and content, discuss perceptions as to how the activities and content may improve health literacy (thereby encouraging participation in play), and how best to present the intervention.
3. To determine the acceptability of the intervention prototype after being tested by the mothers in Soweto.

**2. Methods**

2.1. **Study setting and participants**

This study was nested in the larger Play Love and You (PLAY) study, which was developed with the overriding theme that all that infants need is Play (opportunities for movement and development), Love (responsive and interactive caregiving) and You (the mother herself) [20]. The PLAY study had three aims. The current study was part of the third aim, namely, “To test the efficacy, feasibility and acceptability of providing content, and increasing opportunities for early learning through play to promote literacy”.

In order to develop the intervention, a Community Advisory Group (CAG) was established with the intention of testing the acceptability of the intervention before implementing it in the PLAY Study [20]. Twenty mothers from Soweto with infants aged 0-3 months were recruited in April 2022 to form the CAG. Participants were recruited by approaching women at the taxi rank outside Chris Hani Baragwanath Academic Hospital (CHBAH) to ask whether they would be interested in participating. Mothers were eligible for recruitment if i) they were 18 years of age or older with an infant aged 0-3 months ii) of whom they were the primary caregiver, iii) were living in Soweto for the duration of the study, and agreed to participate for the duration of the study. Mothers were ineligible if infants were born preterm or if either the mother or infant had any disabilities, impairments or disease that would limit movement or impair normal development.

Ethics Statement

Ethical approval was obtained from the University of the Witwatersrand Human Research Ethics Committee (Sub-study M220886 under main study M210846). Written, formal informed consent was obtained from all participants, for participation in the focus group discussions (FGDs) and questionnaires, and for each FGD to be recorded. All participants were reimbursed for travel costs and provided with refreshments.

2.2. **Data collection**

Two rounds of Focus Group Discussions (FDGs) (named “Round 1” and “Round 2”, respectively) were conducted for each participant, approximately two weeks apart in April 2022. All FGDs were conducted at CHBAH in Soweto, Johannesburg by staff trained in qualitative research data collection. Although questions were asked in English, participants could speak in their home language and conversations progressed in the vernacular. This was later transcribed into English for analysis. Lastly, Round 3 of data collection, a telephonic questionnaire, was conducted approximately one week after the completion of the FGDs. All FGDs and telephone calls were audio recorded with the participants’ consent, and transcribed into English verbatim.

**Round 1:**

Round 1 comprised of three FGDs, with eight or nine participants in each FGD, and were conducted over two days. All participants started the session by providing socio-demographic information in a questionnaire. The aim of the Round 1 FGDs was to understand the challenges and barriers that the CAG members experienced when facilitating play with their infants. Participants were given a detailed explanation of what the PLAY study was, including the study’s aims and objectives, themes, contact points between the different arms (intervention and control groups), as well as examples of the types of assessments and interventions that would be included in the study. The theme in the PLAY study that participants were asked to focus on, was around interactive play and promotion of early learning. As a result, discussions were largely around the participants’ understanding of play and development, as well as the effect of their home environment on participating in play with their infants. As part of this discussion, participants were shown a number of photos/ pictures depicting different home environments, which invoked a discussion around which environments enabled or discouraged active play. The stock photos were chosen to show a variety of home environments that would be similar to those found in Soweto, including single roomed homes, shacks and hostels, as well as different settings where children may play e.g. on the side of the road, in a yard, etc. Participants also had a general discussion around the delivery of the intervention, e.g. the preferred means of contact and how often participants should be contacted.

**Round 2:**

Round 2 comprised of two FGDs, with seven to eight participants in each FGD (fifteen in total). Round 2 was primarily focused on a discussion around the acceptability of the play and development aspect of the content to be included in the study intervention. Drawing from the themes which emerged in Round 1 as well as previous research conducted in this same population group, we created a ‘prototype’ or an example of intervention content appropriate for infants aged 0-3 months, which was introduced to the groups in Round 2. This content was in the form of developmentally appropriate activities presented in video format, demonstrations of how to make toys, as well as infographics and other educational material which would be delivered to participants as part of the intervention. Participants were asked to give feedback based on their perceptions as to how the activities and content may improve health literacy, as well as how best to present the intervention. Participants were then asked to complete specific activities at home from the videos (consisting of games to play with baby, trying different tummy time positions), and to make sensory toys demonstrated in person, to determine the potential acceptability of the content in the home setting. They were encouraged to pick a few that they liked and try those, instead of attempting to complete all of them. The videos were sent to the participants via Whatsapp.

**Round 3:**

The participants were then contacted a week later by the study team to complete a questionnaire over the phone. The acceptability questionnaire focused on determining whether the participants found the prototype activities (games presented via video and making of sensory toys) “acceptable” after testing them at home [21]. The acceptability of the activities was determined by asking questions specifically based on the seven component constructs of acceptability by Sekhon et al. [21], which were used to measure acceptability in this study. Each component construct is described further in Fig 1.

**Fig 1: Definition of acceptability and the seven constructs of acceptability** [21].

Fig 2 shows a summary of all three data collection points.

**Fig 2: Summary of data collection**

2.3. **Statistical analysis**

Due to the nature of FDGs, we were able to identify participants during the discussions, however all qualitative data was not identifiable during the analysis. The quantitative data was de-identified at the point of data entry, using the study ID number, so that the researchers could not link the participants to their data. The data used in analysis only had a study ID number, without names or any other identifying information available. The qualitative data was analysed using thematic networks analysis [22]. Meaningful statements and ideas were coded and then grouped into themes. Similar themes were then grouped and placed in thematic networks, which were subsequently summarised and discussed in relation to the original research question. The three transcripts from Round 1, and two transcripts from Round 2 were divided among three researchers who assigned codes to each statement and grouped the codes into themes. The researchers then swapped transcripts to cross-check all the identified themes and codes, which were further checked by a fourth researcher. Thereafter, all researchers met as a group to discuss and refine the themes until no new themes emerged. Means and standard deviations or percentages were calculated on Microsoft Excel and used to describe the demographics of the participants as well as quantitative data from the acceptability questionnaire.

3. **Results**

The average age of the twenty participants was 24 years old, ranging from 18 to 43 years old. Eighty percent of the participants described themselves as single, while the other twenty percent reported themselves to be married, engaged or in a relationship. Seventy percent of the participants were unemployed while thirty percent had some form of employment. The majority (60%) of the mothers had completed their Matric (final year of secondary school), while thirty five percent had not completed secondary school. Only one participant had a tertiary degree. Six of the participants were first time mothers. There was a fairly equal distribution of male and female infants, with half being female, forty five percent male, and one infant whose mother preferred not to say. The youngest baby was new-born and the oldest baby was 5 months old, with the average infant age being 2 months. The time between enrolling participants and the first FGD was just over a month, which explains why some infants were older than the 3 months inclusion criteria.

**Round 1:**

The following eight themes emerged from Round 1 FGDs: 1) Being a mother, 2) Environment, 3) Play and Development, 4) Intervention, 5) Physical Wellbeing, 6) Health Literacy, 7) Breastfeeding, and 8) Mental Health. The Health Literacy, Environment, and Play and Development themes were relevant to this study, while the rest are discussed in papers currently in review. These themes were further divided into subthemes and are discussed below.

**Health Literacy**

**Clinics/ Healthcare workers**

The participants relayed a sense of distrust in the health workers at the clinics. Many reported that they are provided with minimal information, there are long waiting times and health workers can be rude or make new mothers feel ashamed for asking certain questions.

*“No you know at the clinic they don’t explain anything, they just give you Road to Health [booklet]* (*a booklet given to parents by clinic staff when children are born which keeps record of the child’s growth and other key developmental milestones, as well as providing the parent with information on nutrition, loving & bonding, protection of the child including immunisation schedule, basic health care e.g. treating common illnesses and “extra care” for those living with HIV, disabilities, etc) [23] *after giving birth to the baby. You will figure out when you get home when to take the baby in for the three day check-up, and when you have to take them in for six weeks. They don’t explain anything. You just have to give yourself time and read the Road to Health book because at the back they have symptoms that explains the things to watch out for in a child. They don’t explain. You have to sit and read that book.” (Round 1, grp 2).*

**Family/ peers/ other sources**

A few of the participants expressed an interest in receiving more information as they did not have access to family and/ friends who could provide them with advice when it came to their baby’s development. Participants expressed that even when they did have family members involved, they often did not know who to trust and would prefer objective information from a trusted source. However, they did not tend to trust and/ or understand the objective information given to them (such as the Road to Health Booklet), or did not see its relevance, but also did not discuss what/ who they would trust.

*“My mother is there to help me about the baby but I take care of most of the things because my mother loves going out. So most of the time I have to look after the baby so I have to know a lot of things. To clean the belly button and things like that.” (Round 1, grp 2).*

*“I would be happy [*to receive this intervention*], because when you go to the Road to Health [*booklet*], there are some things which are different, if you go to some pages, they talk about SASSA (*an agency that aims to provide comprehensive social security services against vulnerability and poverty) *and I don’t understand what SASSA has to do with Road to Health. (Round 1, grp 2)*

**Environment**

The discussion around the photos of children playing in different settings evoked various thoughts around safety, space and the effect on the child.

**Physical safety**

The participants were quick to point out when an area was not enclosed or when there were many men around, including fathers, as this meant there was potential for the children to be kidnapped or become victims of abuse. There was also mention of children needing to be kept inside or in an enclosed yard to avoid them being involved in Motor Vehicle Accidents.

*“The house is not in a busy part of the township but you need to keep the gate closed as there is a small child. A car can come and drive over the baby if the baby gets out of the yard” (Round 1, Grp 3)*

*“You know, to be honest, these days, you saw how the first picture was* [picture of children playing on the street]*, and then when you check the life that is being lived now and the life from back then* [when the participant was a child], *it is not the same, so it is not going to be easy for my child to relax because fathers these days, they cannot see the difference between a child and someone who is of age, so when they see a child, they don’t care, so I won’t feel free if my child lives in a place like that. A girl especially, not only a girl, because sometimes boys do get raped as well, you understand, I won’t feel happy”. (Round 1, grp 2)*

**Effect on Development**

Many felt that it was important for children to be around other family members such as grandmothers so that they could also look after the child when the mothers were not able to, without the child experiencing separation anxiety. The participants were also aware of how important playing with other children and being around other people was for children’s social development, but there was minimal mention of how the lack of space and outdoor play areas would have an effect on their children’s gross or fine motor play and development.

*“For me, I think it is something good* [for a child to play freely]*, because my first born is a boy. I did not want him to play outside with other kids, I was afraid, like the way I grew up, I was afraid that those things were going to happen to him, so I was keeping him by my side. He was not going to his father, he was with me 24 hours, so I saw that the child is not used to talking, he is not used to doing stuff, he always wants to be with me. Even now, when I say go and visit your place he doesn’t want to, he wants to be with me, so when you close a child up, he can’t communicate with other people. I saw right now at school, to get* [him] *to understand a lot of things, it is hard for him, so I think [you need] to give a child the freedom to play. Yeah, it is a good thing. (Round 1, grp 2)*

**In the house: Occupants and space**

The participants were of differing opinions when it came to the condition of a home and how this may impact the child. Many identified that some of the photos showed unsafe conditions for children, such as exposed wires, potential for injury from kettles/ stoves, lack of running water, etc. Others felt that while certain conditions in their own home were unsanitary or unsafe, they were used to living that way (and taking appropriate precautions) as they had always lived in these conditions, and any accident that may occur as a result of the home environment was due to the parent/s’ negligence.

*“As she says that there is no ideal place where a child can be raised. I feel that the mother can be careless and put water in a dish and not be mindful of the child. If the child falls in the dish and drowns, I feel it would be the mother’s negligence, not that the place is not safe for the child”. (Round 1, Grp 3).*

*Factors hindering play*

The main reasons for respondents not engaging in play with their children included lack of time due to having to go to work, do chores, the perception of being judged by others in the home, and spending time on social media. Some felt that there were no hinderances as they liked to play with their infants, especially as they did not need toys in the first few months.

*“Maybe* [you don’t play] *when you are on the phone and on WhatsApp. Sometimes I am on the phone and when the baby is crying I am like wait a bit you, it is WhatsApp. It is when I am looking at statuses”. (Round 1, grp 2)*

*“The choirs* [others at home] *is what I can say when I think about something that is stopping me, because most of the choirs are looking at me at home.” (Round 1, grp 2)*

“*For me, besides going to work, I don’t think [*there is anything*].” (Round 1, grp 2)*

**Play and Development**

**Activities with infants**

As all of the participants had infants below six months of age, they tended to engage in activities which were appropriate for the age range such as singing and dancing. Some participants also liked to talk to their infants, massage their feet and laugh with them. After seeing a video about ways to engage in “tummy time” with their infants, some participants mentioned that they were not aware that they could put their infant on their tummies in so many different positions.

*“For me, I play with him by singing, and he laughs and enjoys it.” (Round 1, grp 2)*

*“… I play with my child by talking to him. I will talk to him and then sometimes the child shows reactions. He laughs a bit, and then sometimes when I am playing with him, he will look at me but when my other* [child] *is playing with him, he will laugh.” (Round 1, grp 2)*

**Maternal Beliefs**

Many of the participants were aware of what the term “development” meant and some were aware that “milestones” were indicators of how their baby was developing. Some felt that play was good for monitoring how their baby was growing and some had insight into the effect of play on motor development.

*“She develops muscles if she plays and you also see the change in her body”. (Round 1, Grp 1)*

**Round 2:**

During the second round of FGDs, the three themes which emerged from Round 1 (Health Literacy, Environment and Play and Development) were briefly expanded upon. Furthermore, one new theme emerged, namely Acceptability of the intervention prototype content, which was the primary focus of the discussion.

The themes were further divided into subthemes and are discussed below.

**Environment**

**In the house: Occupants and space**

Some participants reported that they live in one room houses where the bedroom and kitchen would be combined, and felt that the space constraints did have an effect on what activities they were able to do with their children. However, those with bigger houses also expressed difficulties with space due to overcrowding.

*“So did you see the games that you showed us, did you see them, they need open space. I said last time, we live in a four room, there are a lot of us. There is no time to do these sort of things. When you are sitting like this, you are sitting like this, and that is the end of it. When you move, you don’t have space anymore. When you go to the bedroom, they follow you. So the time that you get is when you go outside and sit under a tree and sit on the grass, and play. When it is raining, we are not playing we will just be sitting in the house only”. (Round 2, grp 2)*

**Play and Development**

**Maternal Beliefs**

One of the main beliefs discussed in Round 2 was around tummy time and sleep. Most participants believed that infants slept better when they slept on their stomachs, and were not aware of the dangers associated with this.

*“When a baby is sleeping on their tummy, they become better than when they are in other positions and when they’re sleeping on their tummy they sleep”. (Round 2, grp 1).*

**Acceptability**

The main purpose of Round 2 FGDs was to determine the acceptability of the prototype and therefore, this theme was only introduced in Round 2.

The acceptability concept was introduced with a general discussion around the intervention activities, specifically how often the content should be delivered, whether by app or WhatsApp, as well as access to data (or lack thereof) for delivery purposes. All of the participants requested a data free option, and some preferred WhatsApp, while others preferred the idea of using an app. The frequency of the content delivery varied, ranging from daily to weekly and bi-weekly (app content).

Regarding the app content, participants did not mind if the videos were in English, as long as there were subtitles accompanying the audio, moving slowly across the screen. As many of the videos available on the internet are usually North American/ Eurocentric, many of the participants suggested using local videos or having actors who were more representative of the Sowetan Community when it came to their race and culture.

*“It should be maybe it could be a black person. You see?*

*Sometimes we as people, we say these things are done by white people.” (Round 2, Grp 1)*

After being presented with the prototype activities, the participants further discussed their thoughts and perceptions of each activity. Subthemes were then created to align with the seven acceptability component constructs as described in Sekhon et al. [21] and the discussed points were placed into each subtheme, accordingly. Table 1 expands on subthemes and their supporting quotes.

**Table 1: Subthemes and supporting quotes from Round 2 FGDs**

**Round 3**

**Acceptability questionnaire**

The percentage of participants participating in each respective activity is shown in Fig 3. The results of the acceptability questionnaire and participant’s free responses, separated into each component structure are reported below.

**Fig 3: Percentage (%) of participants completing the sensory toys and video activities at home**

One participant was not able to be contacted and therefore only fourteen participants completed the telephonic questionnaire. This participant’s data were excluded from the final quantitative analysis. The number of participants completing each respective activity is represented in Fig 3. The discrepancy in number of mothers completing the activities is due to the mothers only being instructed to pick a few activities to complete at home (i.e. they did not have to complete all of them in the week).

Every participant (100%) reported that they liked playing the games with their infants, largely due to both the baby and participant’s enjoyment, seeing their baby’s development and because it encouraged bonding with their infants.

*“Well I like them a lot because I could see that the baby was developing and I was able to see what he likes and what he thought he did not like and he was smiling and showing me that he was enjoying it. That's why I was able to see that he was enjoying the rolling because for me it seemed like the most uncomfortable one but he was enjoying it. And my son is very picky so I was surprised when he enjoyed rolling. The thing about the games basically you are interacting with your baby and bonding in the time we do these games so I don't think there is anything that I did not like about the games.”* – participant with baby aged 5 months.

*“They make her strong, I can see that she is getting used to them. I am also teaching her the tummy to tummy and I have started doing it a lot. She also likes the games but I can also see that some are helping her with development. She can see and follow sounds, that shows me that there is something that she likes and enjoys. There's a lot that I enjoyed about the games”.* – participant with baby aged 2 months.

Only one participant mentioned that they did not like the sensory square because it was too loud for them even though the baby enjoyed it. Otherwise, the rest of the participants stated that they liked the sensory toys because the infants liked the noise and colours, and holding the square. Other reasons for liking the toys included: using the toys to calm the baby down when they cried, it made the baby happy, and they felt like the baby was learning when using them.

*“They make the baby to be more playful when they touch their toys especially the one with the plastic inside* [sensory square] *it makes the baby so happy” -* participant with baby aged 4 months.

*“I loved everything about it, my baby loves it, I can also see that she can hear it and she wants to hold it” -* participant with baby aged 4 months.

*“I loved the sound that it make because now that she is used to it when she cries it calms her down”.-* participant with baby aged 1 month.

Every participant (100%) felt that the activities were beneficial for them and their baby. Reasons for finding them beneficial included: enjoying the bonding time, both mom and baby (and family) being happy when engaging in the activities, learning and development for both mom and baby, and safety.

*“[*It is beneficial*] because they help me to understand my baby and they help her to develop and to learn many things.” -*– participant with baby aged 2 months.

*“[*It is beneficial*] because when I saw the baby smiling and laughing I was also happy so I am happy that they don't only benefit the baby but myself as well” -* participant with baby aged 1 month.

*“*[It is beneficial] *because the games are safe” -* participant with baby aged 2 months.

Every participant (100%) found the activities easy to do. The participants had varying reasons for finding them easy. These included the fact that they were simple activities and not difficult to understand, because they had seen the videos, that they were bonding with their infants and the baby was enjoying it, and because they had the resources needed for making the sensory square or rice shaker. A couple of participants reported that it was easy because they did not need to buy any toys, which is not something they knew beforehand.

“*I found them easy, they were not as hard as I thought they would be because we only think that playing with the baby requires toys*.” – participant with baby aged 1 month.

*“I can sew, so when I was making the toys I was also enjoying it. She enjoyed all of those and they were easy to do because most of the time these are the games that we play with the baby, it’s just that we did not know that this is what we must do with our children.” -* participant with baby aged 1 month.

*“They were easy because it was just playing with the baby.”* - participant with baby aged 2 months.

Every participant (100%) reported that they understood how to play the games with their infants, however one participant reported that the instructions were not clear. When asked what could be done to make the instructions clearer, the participant responded,

“*Yeah actually I think there should be a person with training to [*show us how to*] play the games sometimes because we forget. So it's easy if we do a practical because other people are slow so it's easy to do a practical for everyone to remember*.” – participant with baby aged 5 months

The majority of the participants (86%) stated that they had time to complete the activities. The remaining two participants were not able to complete the activities due to illness and not knowing that they were required to complete the activities again throughout the week, respectively. When asked what would help them to make time in future, these two participants stated that they would like to be sent the videos again and reminders through SMS.

All of the participants (100%) felt confident that they knew how to do the activities and were doing them correctly. When asked if there was anything that would make them feel even more confident, most of the respondents suggested sending more activities to do, along with instructions on how to do the new activities. Only one participant did not find the toys easy to make.

Of the fourteen participants, eight participants found it very easy to find the resources to make the toys at home, five participants found it easy to find the resources and one participant found it difficult. While most of the participants had the resources at home, one participant needed to buy a packet of chips to add the packet to the sensory square, and another participant needed to buy a needle, which they could both afford.

When it came to how and when the activities were presented, most (97%) of the participants preferred being shown a video instead of it being presented in person, so that they could watch in their own time, slow down and pause the video, and could refer back to it as many times as needed. When asked about how often the participants would like to receive more activities, there were varying responses: five of the participants wanted to receive content once a week, while three participants wanted to receive the content monthly, every two weeks and every day, respectively.

4. **Discussion**

This study aimed to determine the acceptability of a set of activities which would be included in a study intervention for mothers in Soweto. The findings suggest that while many of the participants did enjoy playing with their infants and seeing the impact of play on their child’s development, there were multiple factors which hindered them from playing. These included lack of time or motivation, limited indoor space and lack of safe outdoor space to encourage play, as well as limited availability of information from health workers and/ or limited use of the information available, for example, in the Road to Health Card.

When it came to the mothers’ understanding of play and development, many of the mothers knew about the developmental milestones (although usually only mentioning motor milestones), and perceived their infant reaching these milestones to be a good sign of growth. This is similar to other studies with a similar population group in Soweto, where it was also found that mothers were aware of the different developmental milestones and would use them as a tool for tracking development and progress [15]. While the participants understood the value of play in the younger months, it seemed that the participants were not aware of the importance of play in every aspect of development (e.g. not just physical/ motor development), or of the importance of their own role in promoting play with their infants in the early months. This is similar to a Bangladeshi study which found that rural mothers had basic knowledge about the benefits of play but lacked insight into the importance of it in every aspect of development, particularly the positive impact on cognitive development [24].

A lack of self-efficacy observed in the participants was a finding which was important to consider with regards to planning the intervention. Some of this was due to lack of knowledge and experience but some of it was due to not being in control of the circumstances and environment around them, such as lack of resources and space. This was largely noticeable when many participants showed a sense of excitement and pride in knowing that they could use toys that they had made themselves for their baby’s enjoyment and development, despite their financial constraints. Supporting mothers with education and increased sense of capability, has been shown to have a positive impact on children’s development, seen in a study conducted in India, which found that maternal education and mental health were one of the primary indicators for a positive home environment at 6 and 24 months of age [25].

Furthermore, lack of self-efficacy in facilitating play can be linked to a lack of health literacy in the community, which is often as a result of the mothers’ experiences with the current health system. The participants expressed a strong distrust of the health workers at the clinic (who, in South Africa, are predominantly nurse-led). This distrust is largely as a result of the lack of confidentiality at the clinics and how they are mistreated or made to feel ashamed by the health workers, which results in there being a distrust in the information provided to them by these same health workers. Unfortunately, this is something that is widely reported throughout South Africa. A 2014 report published by Amnesty International found that many of the participants interviewed often avoided attending their local clinic due to fears of lack of privacy and confidentiality. This fear was particularly high if the participant attended a clinic close to where they lived, as the chances of meeting someone they knew, or being treated by a nurse who knew them was much higher [26]. Other studies have reported women feeling insulted and mocked by healthcare workers during birth and ante/postnatal care, women not feeling empowered to ask for information or not receiving information when asked, as well as health workers being unsupportive or unsympathetic to their pain or current needs [27, 28]. Similarly, when discussing access to information and information seeking behaviours, the participants’ distrust of health workers made them hesitant to ask for information around their child’s development, but also unsure of whether any information received could be trusted. Additionally, some participants did not trust information given to them by family or friends, or did not have relatives living with them and therefore relied on information provided by health workers at the clinic. Both scenarios highlight the need for more health literacy in these communities, to allow mothers to be able to decide where to seek information, and to know which sources of information to trust. These findings guided the researchers to provide content which included information on reliable resources online, as well as how to prepare for clinic visits and meetings with Health Professionals.

One of the dominant themes that was repeatedly mentioned, was the impact of the home environment (both inside and outside) on the participants’ movement behaviours and choice of activities for both themselves and their children. Regarding the outside environment, the main concern was around physical safety for the child. This concern is valid given the surroundings in which the participants live. Soweto is an informal settlement or “township” situated on the southwest of Johannesburg. The number of kidnappings reported in South Africa are high, with on average, 23 kidnappings being reported per day between April to September 2021, with approximately 45,8% of these being kidnappings of children [29]. Furthermore, regarding physical safety, a 2019 review of all children attending Red Cross Hospital (a specialist Children’s hospital in Cape Town, South Africa), found that 72% of Road Traffic Injuries were pedestrian injuries, with all of those injured below the age of 4 years, being injured whilst playing close to home [30]. Similar concerns have been voiced by other mothers in the same Sowetan community. Prioreschi et al. [15] found that mothers expressed concerns around their children being hit by cars, being abducted, kidnapped or raped, as well as other concerns which weren’t mentioned in this study but would also be applicable, such as the child hurting themselves or falling into communal toilets. The literature is clear in showing the impact of limited opportunities for movement in home environments (either due to lack of space, overcrowding, etc.) on active play and therefore, motor and cognitive development in infants [31, 32, 33]. As most of the participants reported to having limited space indoors and their environment outside being unsafe, this had major implications in the mother’s ability to encourage active play, both inside and outside. Therefore, in view of these circumstances, it was imperative that for any intervention involving active play to be feasible in this community, limited indoor and outdoor space needed to be taken into account. This resulted in the researchers focusing on increasing health literacy around the value and benefits of play, and specifically how to adapt the home environment, to encourage safe and active play with their infants. However, these activities needed to be developed and tested with the participant group by means of a prototype, to determine their acceptability.

The development and testing of the prototype provided some important insight into how the participants wanted the content to be presented. Overall, the participants were happy to try out the activities at home and did not think of any reason why they would not be able to complete them at home. However, when preparing the activities as part of the prototype, many of the videos or activities did not have a diverse range of ethnicities or socioeconomic groups represented, which the participants did not immediately point out, but when asked further, decided would not be their preference. This was a significant factor which impacted how the intervention activities would be presented in the study, with a focus on including people, environments and resources that were representative of the Soweto community.

When introducing some of the prototype activities, an interesting finding emerged with regards to “tummy time” and sleep. Many of the participants felt that babies slept better in prone position, and were not aware of the associated dangers compared to supine sleeping. This finding is similar to a South African study which found that only 3% of their sample of mothers reported their 6 week old babies were sleeping exclusively in a supine position [34]. The topic was not explored further, as to whether the mothers were encouraged by nursing staff to place their babies in supine when sleeping, however this does further highlight the need for greater levels of health literacy within the community. It also provided further guidance around the need for the inclusion of tummy time information and associated safety precautions in the intervention activities.

When considering the quantitative questionnaire and qualitative data, if using the seven component constructs of acceptability [21], the prototype activities were found to be acceptable to the participants. Therefore, the overall results of the study provided important guidance for the researchers regarding what to consider when creating an acceptable intervention. Firstly, the intervention needed to include simple activities that encouraged age-appropriate play, using very limited resources which were available in the home, for example, making maracas using a plastic spoon, a hollowed-out egg and rice. Secondly, the activities needed to be culturally appropriate for this specific group, for example using singing and dancing activities, to encourage play in younger babies. Thirdly, due to crowded living spaces and limited outside play areas, the activities needed to encourage active play within small spaces. This could be through adapting either the space or activity to be suitable for the mothers’ home environment. Lastly, and perhaps most importantly, due to the limited trust in health workers and lack of reliable information (and support) available to the mothers, the intervention needed to focus on encouraging greater levels of health literacy through provision of information on play and development in the form of infographics and educational videos. For the activities to be feasible in a real-world setting, the results suggested that participation in the activities would be more likely if they helped to develop a sense of self-efficacy in the mothers’, and therefore needed to be simple, with the mothers being able to complete each activity well.

When deciding how the intervention should be delivered, the researchers were guided by the results of the study as well as what was feasible given the constraints of the study (available resources etc.). Findings from a previous study exploring the socio-technical challenges and solutions for designing digital solutions for mothers in Soweto, found that access to WiFi and data were significant barriers for mothers in accessing information digitally [35]. As a result, the researchers decided that intervention content should be provided once a week, through an interactive data free mobile app. All video content was to be made with actors/ models who were representative of Sowetan mothers, usually in English, with subtitles provided where possible. Furthermore, where possible, content would be piloted with the CAG first, allowing for feedback to encourage greater acceptability and feasibility of the content within the real-world setting.

This study provided further insights into Sowetan mothers’ thoughts and perceptions around play and development and what they deemed acceptable activities to be included in an intervention. While most of the activities presented in the sessions and tested at home were found to be acceptable to the participants, the need for context specific and appropriate content was an important concept that needed to frame all future intervention activities. This study has certain limitations, which the researchers tried to mitigate wherever possible. As this is small sample, the findings of this study could potentially not be generalisable to other population groups or settings. A common risk when using focus group discussions, is the risk of the discussion being led based on the researchers’ agenda, or the agenda of specific participants in the group. This risk was minimised by having a trained facilitator who was external to the project and an expert in dealing with group dynamics to allow for equal contribution from each participant. As the author is an outsider to the study population community, with their own ideas and presuppositions, as well as this study being completed as part of a doctoral degree, it is expected that some interpreter bias was present. However, where possible, interpreter bias was minimised by for example, having three researchers code and re-code the data as described in the methods. A fourth researcher then consolidated the codes and themes to allow for further independent analysis before write up of the findings began.

5. **Conclusion**

In conclusion, this study has provided insight into the acceptability of an intervention designed to improve health literacy around developmentally appropriate play during infancy, with a community advisory group of mothers in the Soweto community. Further insight was gained into how mothers perceive play, and how they are often not aware of their agency in impacting their infants’ development. Barriers to play, such as limited access to safe spaces, overcrowding and limited resources were explored as well as factors contributing to the participants’ level of health literacy and how this impacts their information seeking behaviours. The findings presented in this study have advised the development of an intervention in the form of an interactive mobile app and could provide further insight and guidance for developing future interventions in low income settings.

6. **Acknowledgements**

The researchers would like to acknowledge the PLAY study research team for their assistance in collecting the questionnaire data and providing administrative support in the Focus Group Discussions. We would also like to acknowledge Lebo Motlhatlhedi for conducting the Focus Group Discussions.

7. **References**

[1]	Scientific Council on the Developing Child N, National Forum on Early Childhood Policy. The foundations of lifelong health are Built in early childhood. 2010.

[2]	Crumbley CA, Ledoux TA, Johnston CA. Physical Activity During Early Childhood: The Importance of Parental Modeling. Am J Lifestyle Med 2020;14:32–5. https://doi.org/10.1177/1559827619880513.

[3]	Trawick-Smith J. The Physical Play and Motor Development of Young Children: A Review of Literature and Implications for Practice. 2014.

[4]	Draper CE, Tomaz SA, Biersteker L, Cook CJ, Couper J, De Milander M, et al. The South African 24-hour movement guidelines for birth to 5 years: An integration of physical activity, sitting behavior, screen time, and sleep. J Phys Act Health 2020;17:109–19. https://doi.org/10.1123/jpah.2019-0187.

[5]	Ginsburg KR, Shifrin DL, Broughton DD, Dreyer BP, Milteer RM, Mulligan DA, et al. The importance of play in promoting healthy child development and maintaining strong parent-child bonds. Pediatrics 2007;119:182–91. https://doi.org/10.1542/peds.2006-2697.

[6]	Venter L. A systems perspective on early childhood development education in South Africa. International Journal of Child Care and Education Policy 2022;16. https://doi.org/10.1186/s40723-022-00100-5.

[7]	Jeffery C. Tanner, Tara Candland, Whitney S. Odden. Later Impacts of Early Childhood Interventions: A Systematic Review. 2015.

[8]	UNICEF. Learning through play Strengthening learning through play in early childhood education programmes in support of 2 Learning through play Strengthening learning through play in early childhood education programmes. 2018.

[9]	Piek JP, Dawson L, Smith LM, Gasson N. The role of early fine and gross motor development on later motor and cognitive ability. Hum Mov Sci 2008;27:668–81. https://doi.org/10.1016/j.humov.2007.11.002.

[10]	Erhardt RP, Meade V. Improving handwriting without teaching handwriting: The consultative clinical reasoning process. Aust Occup Ther J 2005;52:199–210. https://doi.org/10.1111/j.1440-1630.2005.00505.x.

[11]	UNICEF, WHO, World Bank Group. Levels and trends in child malnutrition. 2021.

[12]	Klingberg S, Draper CE, Micklesfield LK, Benjamin-Neelon SE, van Sluijs EMF. Childhood obesity prevention in africa: A systematic review of intervention effectiveness and implementation. Int J Environ Res Public Health 2019;16. https://doi.org/10.3390/ijerph16071212.

[13]	Statistics SA. Income dynamics and poverty statutes of households in South Africa. 2015.

[14]	STATS SA. General Household Survey (2021). 2022.

[15]	Prioreschi A, Wrottesley SV, Slemming W, Cohen E, Norris SA. A qualitative study reporting maternal perceptions of the importance of play for healthy growth and development in the first two years of life. BMC Pediatr 2020;20. https://doi.org/10.1186/s12887-020-02321-4.

[16]	WHO. Improving Early Childhood : WHO Guideline. 2020.

[17]	WHO. Health literacy development for the prevention and control of noncommunicable diseases Volume 1. 2022.

[18]	WHO. Health literacy development for the prevention and control of noncommunicable diseases. 2022.

[19]	Hall K. Children living with parents. University of Cape Town Children Count 2019.

[20]	Prioreschi A, Pearson R, Richter L, Bennin F, Theunissen H, Cantrell SJ, et al. Protocol for the PLAY Study: a randomised controlled trial of an intervention to improve infant development by encouraging maternal self-efficacy using behavioural feedback. BMJ Open 2023;13:e064976. https://doi.org/10.1136/bmjopen-2022-064976.

[21]	Sekhon M, Cartwright M, Francis JJ. Acceptability of healthcare interventions: An overview of reviews and development of a theoretical framework. BMC Health Serv Res 2017;17. https://doi.org/10.1186/s12913-017-2031-8.

[22]	Attride-Stirling J. Thematic networks: an analytic tool for qualitative research. Qualitative Research 2001;1:385–405. https://doi.org/10.1177/146879410100100307.

[23]	SA Department of Health. Road to Health Booklet. 2022.

[24]	Sharif S. Rural Mothers’ Perceptions of Play in Children’s Learning and Development in Bangladesh. 2016.

[25]	Koshy B, Karthikeyan A, Bose A, Roshan R, Ramanujam K, Mohan VR, et al. Home environment: Short-term trends and predictors in early childhood from an Indian community birth cohort. Child Care Health Dev 2021;47:336–48. https://doi.org/10.1111/cch.12846.

[26]	Amnesty International. Struggle for maternal health: Barriers to antenatal care in South Africa. 2014.

[27]	Silal SP, Penn-Kekana L, Harris B, Birch S, Mcintyre D. Exploring inequalities in access to and use of maternal health services in South Africa. vol. 12. 2012.

[28]	Pickles C. Eliminating abusive ‘care’: A criminal law response to obstetric violence in South Africa. South African Crime Quarterly 2015;54:5. https://doi.org/10.4314/sacq.v54i1.1.

[29]	Institute for Security Studies. Who gets kidnapped in SA? 2022.

[30]	Childsafe SA. 2019 Childhood Injury Data Report. n.d.

[31]	Liang CY, Chen CL, Chou LT, Chang CJ, Chung CY. Effects of home environment on changes in the motor development for typical-development infants and toddlers. Ann Phys Rehabil Med 2018;61:e554–5. https://doi.org/10.1016/j.rehab.2018.05.1291.

[32]	Miquelote AF, Santos DCC, Caçola PM, Montebelo MI de L, Gabbard C. Effect of the home environment on motor and cognitive behavior of infants. Infant Behav Dev 2012;35:329–34. https://doi.org/10.1016/j.infbeh.2012.02.002.

[33]	Niemistö D, Finni T, Haapala EA, Cantell M, Korhonen E, Sääkslahti A. Environmental correlates of motor competence in children-the skilled kids study. Int J Environ Res Public Health 2019;16. https://doi.org/10.3390/ijerph16111989.

[34]	Russell D, Kriel H, Joubert G, Goosen Y. Prone positioning and motor development in the first 6 weeks of life. South African Journal of Occupational Therapy 2009;39.

[35]	Klingberg S, Motlhatlhedi M, Mabena G, Mooki T, Verdezoto N, Densmore M, et al. “Must you make an app?” A qualitative exploration of socio-technical challenges and opportunities for designing digital maternal and child health solutions in Soweto, South Africa. PLOS Global Public Health 2022;2:e0001280. https://doi.org/10.1371/journal.pgph.0001280.

8. **Supporting information**

**S1 Fig 1. Definition of acceptability and the seven constructs of acceptability.**

**S2 Fig 2. Summary of data collection**

**S3 Fig 3. Percentage (%) of participants completing the sensory toys and video activities at home**

**S1 Table 1. Subthemes and supporting quotes from Round 2 FGDs**

9. **Funding**

This work was supported by the National Institute for Health Research (NIHR) (using the UK’s Official Development Assistance (ODA) Funding) and Wellcome [222007/Z/20/Z] under the NIHR-Wellcome Partnership for Global Health Research. The views expressed are those of the authors and not necessarily those of Wellcome, the NIHR or the Department of Health and Social Care.
