# Supplementary material for: Determining the perceived acceptability of an intervention designed to improve health literacy around developmentally appropriate play during infancy, with a community advisory group of mothers, in Soweto, South Africa
Source: PLOS Glob Public Health. 2024 Aug 29;4(8):e0002233. doi: 10.1371/journal.pgph.0002233 (PMC11361429; doi:10.1371/journal.pgph.0002233)
Supplement: Attachment — Submitted filename: Response to Reviewers .docx [file pgph.0002233.s009.docx]

Dear Editor,

Kindly see the response to the reviewer and editor’s comments below, as requested.

Kind regards,

Fiona Bennin

| **Reviewer’s comment** | **Response** | **Line number** |
| --- | --- | --- |
| Line 63 – what are the negative effects that may be experienced? | The introduction has been trimmed down and as a result, this paragraph has been deleted to be more succinct. | N/A |
| Line 94-95 split the two ideas | Have removed stunting, as conversation was moved to beginning of intro and stunting did not fit in to what is being discussed. Therefore no need to split the two ideas | Line 61-62 |
| Line 97 – there is an updated census please check and use latest statistics | The statistic from the census and accompanying information has been removed to be more succinct. | N/A |
| Line 105 – SA is a low-middle-income-setting | Have changed to low-middle income setting | Line 102 |
| *Methods* |  |  |
| - Were participants in all rounds of the focus group discussions the same as round 1 or were there different participants for each round? | An explanation has been added to describe how all participants in Round 2 and 3 had been in Round 1 and were asked to return (some did not return, hence Round 2 and 3 having fewer participants than Round 1) | Line 306-307 |
| Line 249-250 – this sentence is unclear what is meant by this? Is it referring to the anonymity of participants? Please be more clear this is difficult to understand | Yes, this is referring to the anonymity of the participants. A sentence has been added to explain that anonymity could not be maintained. | Line 359 |
| More information about the participants should be given what was the exact sample for each round of data collection. Please be specific | Further breakdown of the exact sample sizes has been added for each Round. | Line 284-285; 306-308; 333 |
| *Findings* |  |  |
| Please provide insight into the 8 themes identified in the first round | A description of each theme has been provided. | Line 398-410 |
| What is meant by the environment theme each the theme above has an introduction please be consistent throughout the themes and provide insight as to what is meant by the environment theme | The environment theme has been described in greater detail (as above) | Lines 339-401 |
| What were the themes that were identified in round two? Please help the reader through the findings so that they know which themes will be discussed in each round | Round 2 has now merged with Round 1 but an explanation of the 3 themes that were discussed again (from Round 1) as well as a new theme (acceptability) is included. | Lines 412-416 |
| Round 2 needs more granular quotes to help the reader understand what the theme is about and what the perspectives of the caregivers were especially the acceptability component. | The table exploring each construct and related quotes has now been included in the main text (previously was added as a supplementary file). This provides more granular quotes related to each acceptability component. | Line 773-775 |
| This theme provides limited information about acceptability please provide more insight as to what is acceptable according to the participants | More information has been provided in Table 1, now provided (as above). | Line 761-762 |
| Round 3 Provide information as to what the acceptability questionnaire was about | As this has been added to the methods (see below), this hasn’t been added again in results, to avoid repetition. | Line 334-336; 340-341 |
| In the methods, please add a section to highlight what the questionnaire is and what types of questions were asked so that the reader can understand what the questionnaire is about. | Examples of questions have been added to the methods and more info on what the questions focused on. | Line 334-336; 340-341 |
| Line 507-rephrase one of the participants was not contactable | Changed to, “one participant was not contactable…” | Line 791 |
| Overall, 14 participants are quite a small number to draw conclusions as to whether the intervention is acceptable (n=14) participants are not statistically significant. Could the authors please provide insight as to why only 14 participants were included in the questionnaire? | We agree that one cannot infer statistical significance from such a small sample and have therefore focused more on discussing the qualitative results and how these do/ do not support acceptability of the intervention. Logistically, we could only include the participants from the initial Focus Groups in our questionnaire sample, which is why only 14 were included. Hopefully this is clearer now that we have made it clearer that the same participants were included throughout all 3 Rounds of data collection. | Line 306-307; 333 |
| The quantitative component is lacking, and I would suggest highlighting the descriptive statistics and expanding on them in the text.  FROM EDITOR: You can ignore the comment about the quantitative analysis. The focus should be on the qualitative results. A sample size of 14 is too small to make any general inference and the descriptive analysis you have conducted is already a stretch. Instead, I suggest using demographic information from these respondents to provide some indication of whether you think the intervention is likely to be acceptable to the wider population (this can be in the discussion section where you talk about the limitations | We agree that we should focus on the qualitative results and have only described the demographics and acceptability responses in order to determine whether the women we tested found the activities acceptable or not.  Instead of using the standard deviation we have just used the median and range to describe the data for the demographics and questionnaire. | Line 371-373; 381- 391 |
| The discussion should not present any new findings rather just provide a discussion and highlight the similarities and/or difference across the literature. Highlight the strengths and limitations of the study, policy implications and future recommendations | No new findings have been added, only discussed with relation to other literature as suggested. Have added policy recommendation at the end of the conclusion. | Lines 1165-1166 |
| Findings and discussion need more work to help the reader fully understand the findings. | Subheadings have been added to guide the reader through the different discussion points, linked to each “round” of results. Edits have been made to provide more nuance in the discussion. | Lines 884 - 1125 |
| More information about the analysis is required | This has hopefully been addressed by removing the quantitative component and focussing more on the qualitative analysis. | Lines 884 – 1125; 358-373 |
| **Editor’s comments (major)** | **Response** | **Line number/ page** |
| The Introduction section can be streamlined significantly. Perhaps the key messages in the paragraphs can be: 1) sedentary lifestyles and associated poor health among children is on the rise, 2) interventions that facilitate movements at home are critically needed, 3) the objectives of the study. The part about findings from previous studies can be moved to the discussion section. Also, there is a fair amount of repetition here that you can remove/shorten without losing substance. | Have edited the introduction into the three main sections, as suggested. | Lines 58-148 |
| Please consider editing the entire manuscript for clarity and brevity. I’ve made only a few minor edits for your consideration. | Have done our best to edit the highlighted corrections as well as other longer or vague sentences. | Whole body of text |
| MAJOR: Please provide a rationale for why you focused on children in this age group. Children below 3 months don’t move much, except when their parents do. | Have added rationale in the introduction as well as in the methods. There is a plethora of evidence to show that is imperative that early movement behaviours start from birth as these behaviours are associated with positive outcomes later in life. | Lines 171-174 |
| Not sure if this is considered an eligibility criteria. Fine to remove it. | Have removed it. | Line 167 |
| Child’s? (I am guessing mothers were eligible even if they themselves had mobility-related impairments) | Correct, the criteria only mention the infant - “if the infant had any disabilities, impairments or disease that would limit movement or impair normal development | Lines 170-171 |
| And translated to English if they responded in a different language? | Correct. Have edited with this additional information. | Line 189 |
| Not sure how it worked, since you have 20 participants. Did some participants attend multiple sessions?  May be provide a table in the appendix with the number of participants in each FGD. | Have added further breakdown for each FGD as well as an appendix 1. | Lines 2021-202 |
| ?? Aren’t all mothers receiving the intervention here? | As this was the CAG, this group of mothers would not be receiving the trial intervention but only received parts of the intervention to test for feasibility and acceptability. Have added, “of the trial” to make it clear it’s regarding the trial and not this group. | Line 207 |
| Ideally, these should be included as online appendix so future researchers can also benefit from them. | Have included as a supplementary file that can be used as an online appendix 2 | Line 213 |
| MAJOR: At the time of revision, please submit this as a supplementary file. Please also describe this in greater detail here. | Have added more detail about the content as well as a supplementary file | Line 235 |
| Please include a sentence or two here mentioning the 7 components. | Have included a sentence describing the seven components | Lines 268-270 |
| I’d just call it Data Analysis since most analysis is qualitative here. | Have changed to “Data Analysis” | Line 278 |
| Not clear how this or the rest of the paragraph relates to play, the focus of the study. | Have added a sentence explaining that they have limited information on play and development. | Lines 350-351 |
| Also not clear how this relates to play. Please clarify. | It came out in the results that the participants do not have access to information both from the health system or informally from their peers and family, which has been a large motivator for why additional information was provided in the intervention, with the aim of improving health literacy in the area of play and development | Lines 376-379 |
| Instead of presenting the results by round, consider doing it by theme. Might be easier to follow that way, and you will not end up repeating the same subtopics. | Have combined Round 1 and 2, so that the themes are not repeated, as suggested. | Lines 315-712 |
| Fine, but worth summarizing the takeaway messages here in the text for each component. (Also, isn’t this S1 Table 1? Please move the table to the main text, as the findings are on the paper’s main research question!) | Have included the table as it should be included, and, because it has takeaway messages for each component as requested | Lines 705-709 |
| This seems to be saying that infographics and videos sent through whatsapp are better than direct communication from health workers. I would tone it down because your data do not support that at all. In fact, the whole thing about trust in the health system is a bit of a digression here, and you wouldn’t lose much by way of content if you decided to remove it (both here and in the results section above). | While we agree that it’s not a major factor, the distrust in the health system does relate to health literacy, as we know from other literature that the mothers don’t ask questions or request what they need and therefore do not have the information required to make informed choices about their children’s health. Adding the content in the intervention would be an additional support. However, the authors have toned it down and made it clear that it is not instead of, but working with the current resources, to help improve health literacy | Lines 1015-1019 |
| How were they selected in this study? | This article was written before the content was completed, and in that time, this detail was changed to only having animated characters | Line 1027 |
| This is too general to be inferred from this study. Consider removing it. Shouldn’t ALL interventions be tested for acceptability and feasibility anyway? | Have removed | Line 1029 |
| Additional limitations not discussed here: 1) responses self-reported by study participants. This doesn’t matter for most findings here, but the ones about how frequently mothers play with children, should be taken with caution. 2) we don’t know how actively mothers engaged with the intervention material (unless you collected are on how frequently the videos you sent were watched, or if they were watched at all). | Added as suggested | Lines 1080-1084 |
| But do you have a sense of how similar this area is (or the group of women recruited are, based on their demographics) to the rest of the population? | More text has been added to show that these findings could be generalisable to this population despite the small sample size, due to the demographic data being similar to the data found in other studies with larger sample sizes. | Lines 1084-1089 |

-
