# Supplementary material for: Determining the perceived acceptability of an intervention designed to improve health literacy around developmentally appropriate play during infancy, with a community advisory group of mothers, in Soweto, South Africa
Source: PLOS Glob Public Health. 2024 Aug 29;4(8):e0002233. doi: 10.1371/journal.pgph.0002233 (PMC11361429; doi:10.1371/journal.pgph.0002233)
Supplement: Attachment — Submitted filename: Response to Reviewers.docx [file pgph.0002233.s010.docx]

Dear Editor,

Thank you for your feedback. Kindly see the response to your comments below:

| **Editor/ Reviewer comments** | **Authors’ response** |
| --- | --- |
| Comments from Editor:  Please review your reference list to ensure that it is complete and correct. If you have cited papers that have been retracted, please include the rationale for doing so in the manuscript text, or remove these references and replace them with relevant current references. Any changes to the reference list should be mentioned in the rebuttal letter that accompanies your revised manuscript. If you need to cite a retracted article, indicate the article’s retracted status in the References list and also include a citation and full reference for the retraction notice. | The reference list has been reviewed again and only reference number 1 has been edited to be correct.  A website URL or DOI has been included for all those that had this missing. This includes references number: 1, 2, 5, 9, 10, 14, 15, 17, 18, 20, 21 and 25.  None of the references have been retracted. |
| Additional Editor Comments (if provided):  Please address the comments from the reviewer and resubmit. The comments seem straightforward to address. Feel free to ignore the comment about quantitative analysis, as I am aware of the limitations of that analysis. Please read the manuscript thoroughly for flow and clarity. | No comments have been included from the reviewer in the email that was received from the editor. This was followed up and the journal reported that, “It appears that the Academic Editor did not send the most recent version of your manuscript out for external reviews, but rather arrived at this decision based on his or her own expertise.... In this case, there are no review comments to provide”.  The comments regarding the quantitative analysis were addressed in our previous manuscript but if there are still concerns, kindly let us know.  We have read through the manuscript again for flow and clarity and made a few minor grammatical changes throughout the manuscript. |
| Reviewers' comments: None | N/A – as above |

Given the points above, we think that all editorial concerns have now been addressed. We hope that we have understood the outstanding issues but if there are any other points that we have missed, we are happy to edit as required.

Kind regards,

Fiona Bennin

-
