# Supplementary material for: Determining the perceived acceptability of an intervention designed to improve health literacy around developmentally appropriate play during infancy, with a community advisory group of mothers, in Soweto, South Africa
Source: PLOS Glob Public Health. 2024 Aug 29;4(8):e0002233. doi: 10.1371/journal.pgph.0002233 (PMC11361429; doi:10.1371/journal.pgph.0002233)
Supplement: Attachment — Submitted filename: Response to Reviewers.docx [file pgph.0002233.s011.docx]

| **Reviewer comment/ request:** | **Response (line numbers are from ‘clean’ manuscript)** |
| --- | --- |
| Check the language and sentence structure throughout the manuscript. Overall, the discussion can be shortened and at times there are information that has not be touched on in the findings section. What are the strengths of the study? I am also missing the recommendation for policy, practice and research now that the findings are there what should be done next? | The manuscript has been edited again for more concise language and sentence structure. The discussion has been tightened. The strengths and recommendations have been further elaborated on in the last paragraph of the discussion and the end of the conclusion. |
| Line 185 – change the N to n | Changed as requested (line 184) |
| Line 190-193 – please make these two sentences as it is too long and difficult to follow | Two sentences have been created (line 189 – 193). |
| Line 196 – 200 – Please separate sentence as it becomes a bit difficult for the reader to follow. For example, We asked participants to engage and complete 3 specific activities at home. The first activity was based on the videos which consisted of (). The second activity was for caregivers to make a sensory toy which was demonstrated in person. The last activity was to determine the potential acceptability of the content in the home setting from the perspective of the caregivers. | This has been separated into 4 sentences (line 196-201). |
| Line 226 – When interviewing participants, the identities will always be known to us. I think you can delete the sentence and highlight that participants identities were protected by assigning a unique identifier/pseudonym to the participant. | Sentence has been deleted and it is highlighted that all participants’ identities were protected by assigning unique identifiers to participants (lines 226-227). |
| Results Line 244 – 256 stay consistent with whether you are going to write out the percentage in words or use numerical values to show the percentages. | All percentages now are written out as the word “percentage” instead of “%”, for consistency (line 247). |
| Line 293 – Make a footnote explaining the road to health as it disrupts the flow of the quote. | A footnote has been added.  A footnote has been added for the terms “Road to Health booklet” and SASSA (following line 313) |
| Line 335 – motor vehicle accidents should not be capitalized | Changed to lowercase (line 327) |
| Line 341 – 345 – This quote can be shortened as most of the information is not relevant the description of the theme. | Quote has been shortened for relevance (lines 333-336) |
| Line 351- 353 – you can make this into two different sentence to make it easier to follow | This has been separated into two sentences (line 341). |
| Line 359-367 this quote can be shortened and only highlight the most prominent aspects of the effects on development. | Quote has been shortened to highlight the effect on development (line 348-351). |
| Line 411 – you can remove this quote | Quote has been removed |
| line 606 – stay consistent with terms you in pervious sections you mention road to health booklet and in this line you use road to health card | The word “booklet” has now been inserted throughout the text for consistency (line 587) |
| line 633 – 635 provide a reference | This has been taken out as it was an opinion of the first author. |
| References | References have been checked and updated DOIs have now been included. |
